# Definitive intensity modulated radiotherapy in locally advanced hypopharygeal and laryngeal squamous cell carcinoma: mature treatment results and patterns of locoregional failure

**DOI:** 10.1186/s13014-014-0323-2

**Published:** 2015-01-17

**Authors:** Andreas Geretschläger, Beat Bojaxhiu, Alan Dal Pra, Dominic Leiser, Michael Schmücking, Andreas Arnold, Pirus Ghadjar, Daniel M Aebersold

**Affiliations:** Department of Radiation Oncology, Inselspital, Bern University Hospital and University of Bern, Freiburgstrasse, 3010 Bern, Switzerland; Department of Otorhinolaryngology, Bern University Hospital, Freiburgstrasse, 3010 Bern, Switzerland

**Keywords:** Hypoharyngeal cancer, Laryngeal cancer, IMRT, Patterns of failure, Radiotherapy

## Abstract

**Purpose:**

To assess clinical outcomes and patterns of loco-regional failure (LRF) in relation to clinical target volumes (CTV) in patients with locally advanced hypopharyngeal and laryngeal squamous cell carcinoma (HL-SCC) treated with definitive intensity modulated radiotherapy (IMRT) and concurrent systemic therapy.

**Methods:**

Data from HL-SCC patients treated from 2007 to 2010 were retrospectively evaluated. Primary endpoint was loco-regional control (LRC). Secondary endpoints included local (LC) and regional (RC) controls, distant metastasis free survival (DMFS), laryngectomy free survival (LFS), overall survival (OS), and acute and late toxicities. Time-to-event endpoints were estimated using Kaplan-Meier method, and univariate and multivariate analyses were performed using Cox proportional hazards models. Recurrent gross tumor volume (RTV) on post-treatment diagnostic imaging was analyzed in relation to corresponding CTV (in-volume, > 95% of RTV inside CTV; marginal, 20–95% inside CTV; out-volume, < 20% inside CTV).

**Results:**

Fifty patients (stage III: 14, IVa: 33, IVb: 3) completed treatment and were included in the analysis (median follow-up of 4.2 years). Three-year LRC, DMFS and overall survival (OS) were 77%, 96% and 63%, respectively. Grade 2 and 3 acute toxicity were 38% and 62%, respectively; grade 2 and 3 late toxicity were 23% and 15%, respectively. We identified 10 patients with LRF (8 local, 1 regional, 1 local + regional). Six out of 10 RTVs were fully included in both elective and high-dose CTVs, and 4 RTVs were marginal to the high-dose CTVs.

**Conclusion:**

The treatment of locally advanced HL-SCC with definitive IMRT and concurrent systemic therapy provides good LRC rates with acceptable toxicity profile. Nevertheless, the analysis of LRFs in relation to CTVs showed in-volume relapses to be the major mode of recurrence indicating that novel strategies to overcome radioresistance are required.

## Background

Radical surgical treatment of locally advanced squamous cell carcinoma of the hypopharynx or larynx (HL-SCC) often requires total laryngectomy (TL). Landmark clinical trials for laryngeal [1] and hypopharyngeal cancers [2] have shown that organ preserving treatments such as induction chemotherapy followed by radiotherapy (RT) are non-inferior to surgical treatment followed by RT. Subsequently, concurrent chemoradiation further improved locoregional control (LRC) in comparison to sequential induction chemotherapy and RT in the RTOG 91-11 trial [3,4] and is since regarded as standard treatment for locally advanced HL-SCC.

In the last decade intensity-modulated radiotherapy (IMRT) has replaced 3D conformal RT for definitive treatment of locally advanced head-and-neck cancers due to the highly conformal dose distribution with steep gradients towards the surrounding healthy tissues thereby sparing unwanted dose to organs at risk [5,6]. In a prospective randomized trial, parotid-sparing IMRT significantly reduced xerostomia compared to 3D conformal RT [7]. Of note, a tight conformal dose distribution might instead increase the likelihood of geographical miss and locoregional failure (LRF) [8]. Patients with locally advanced HL-SCC have a high probability of both clinically evident and occult lymph node metastasis and subclinical mucosal tumor spread. IMRT treatment should be accompanied by a rigorous quality assurance program in order to provide early identification and analysis of locoregional treatment failures. Having introduced IMRT in our institutional clinical practice in 2002, we reevaluated and adapted our guidelines for target volume definition for HL-SCC in 2007 drawing upon our own experience as well as early publications on IMRT in head-and-neck cancers [9-11]. With the aim of further improving treatment results through continuous analysis of our LRF patterns we have retrospectively analyzed mature clinical outcomes and toxicity patterns of a cohort of patients treated from 2007 onwards according to these standards.

## Methods

### Patient selection

Patients with locally advanced HL-SCC [American Joint Committee on Cancer (AJCC) stage III or IV] treated with curative IMRT treatment between January 2007 and December 2010 at the Department of Radiation Oncology, Inselspital, Bern University Hospital were retrospectively assessed. Ineligibility criteria included patients older than 85 years, initial Karnofsky Performance Status (KPS) less than 60%, history of another malignancy within 5 years of diagnosis, prior RT to the head and neck, histology other than squamous cell carcinoma and distant metastatic disease. Patients who underwent radical surgical procedures to the primary tumor were excluded, but functional tumor debulking and/or primary neck dissections (ND) were allowed. Patients who did not reach the prescribed RT dose or did not finish treatment within 60 days since delivery of the first RT fraction were excluded. Living patients with documented follow-up of less than one year were also excluded from analysis. This study was approved by the local research ethics committee and the Swiss Federal Office of Public Health (FOPH) (035.0001-90).

### Treatment

All cases were presented at the weekly institutional interdisciplinary head-and-neck tumour board. After completion of staging examinations and final TNM staging (AJCC), selection of treatment modalities and treatment sequencing were defined. Pre-treatment staging involved laryngoscopy, measurement of the tumour, and high-resolution computed tomographic (CT) scanning of the primary tumour and the neck with or without magnetic resonance imaging (MRI). Prior to 2008, imaging for systemic staging was performed as clinically indicated; and from 2008 onwards positron emission tomography with 2-deoxy-2-[fluorine-18]fluoro-D-glucose integrated with computed tomography (18F-FDG PET/CT) was regularly used in the staging of locally advanced cases to rule out distant metastasis, synchronous malignancies, and gain additional information on lymph node metastasis, especially on retropharyngeal (RP) node status. All patients were referred to dental evaluation before start of RT. Prophylactic feeding tube insertion via gastrostomy was recommended to most of the patients.

### Radiotherapy

For treatment planning a dedicated high-resolution CT scan with 3 mm slices and intravenous contrast was used. Patients were immobilized in the supine position using a thermoplastic mask covering head and shoulders. No bite bock was used to facilitate dorsal hyperflexion of the cervical spine. Patients were additionally instructed to keep the tongue extended during treatment if possible. All visible surgical scars were marked with flexible wires if a neck dissection had been performed. All target volumes and organs at risk were contoured on the planning CT scan. Image fusion of diagnostic MRI and/or PET-CT with the planning CT was performed if cervical spine flexion was similar resulting in an acceptable matching distortion. Gross tumor volume (GTV) consisted of the primary tumor (or residual tumor after debulking in two patients) and pathologic lymph nodes as clinically diagnosed and visualized on all available imaging. High-risk CTV72 was derived from the GTV with an additional isotropic margin of 5 – 12 mm. Only in patients with prior ND, a high-risk CTV66 was defined as the levels of the original gross involved nodes harbouring extracapsular extension (ECE), as reconstructed from the preoperative imaging, with an additional isotropic margin of 5 – 15 mm. In nodal stages pN2a and pN3 we generally assumed presence of ECE even if not explicitly stated in the histopathologic report. In cases of reported intraoperative lymph node spillage the complete area of neck dissection was included in the CTV66.

In patients with ND and nodal stages pN1, pN2b, pN2c without ECE, the standard risk CTV54 included all dissected neck levels and the surgically manipulated area as proposed by Gregoire et al. [12] and all non-dissected elective lymph node levels bilaterally. In patients without ND, CTV54 included all elective lymph node levels bilaterally. The definition of elective nodal target volumes in respect to size and location of the primary tumor followed the recommendations proposed by Eisbruch et al. [13] and consensus guidelines [14] with minor adjustments. In clinical or pathological nodal stages N1, N2a and N2b, we did not include the cranial half of contralateral level II to spare contralateral parotid gland neither caudal half of contralateral level IV. When present, the area of tracheostomy was fully included in the CTV54. In cases of ipsilateral level II involvement, CTV54 extended cranially to the base of skull on the ipsilateral side including the retro-styloid space [11]. If ipsilateral level II was not involved in nodal stage N1, N2a, N2b, we also considered sparing the cranial half of ipsilateral level II. In the absence of metastatic RP nodes after diagnostic imaging with MRI and PET-CT, elective coverage of RP were limited to 3-4 cm cranially to the GTV.

All prescribed CTV margins were manually adapted to account for anatomical barriers such as thyroid and cricoid cartilages, hyoid, mandibular or vertebral bones, skin and air. The resulting CTVs were finally expanded to planning target volumes (PTVs) by adding a symmetric 3 mm margin for setup error compensation. In a final adjustment PTVs were set back from the skin surface 3 mm to allow for dose build-up except where the skin was deemed to be at risk of microscopic disease. Instead of using a skin flap in these cases we tried to optimize dose build-up by putting more weight on tangential beam directions accepting minor under-dosage. Prescribed doses to PTV72, PTV66 and PTV54 were 72 Gy, 66 Gy and 54 Gy, respectively. All PTVs were treated sequentially in a “shrinking-volume” technique with a fractionation of 5 times per week, 2Gy per fraction, resulting in two or three treatment plans per patient. All PTVs were comprehensively covered in one IMRT plan without field junctions. The total dose was prescribed to the median dose (D50%) of the PTV in accordance with the ICRU report 83. Pre-treatment setup imaging was performed daily. All treatment plans were contoured and calculated using Eclipse treatment planning system (Varian Medical Systems, Palo Alto, CA).

### Concomitant systemic therapy

The standard concomitant therapy consisted of cisplatin 100mg/m2 day 1 in three-week intervals for all patients. In few cases of induction chemotherapy, cisplatin, docetaxel and 5-fluorouracil were used. Patients not deemed medically fit for cisplatin chemotherapy because of pre-existing co-morbidities were evaluated for weekly treatment with monoclonal antibody cetuximab [15] or carboplatin three weekly. Patients who did not tolerate the toxicity of cisplatin treatment were switched to either carboplatin as described or to low dose carboplatin weekly or to cetuximab at the discretion of the medical oncologist.

### Assessments and evaluations

After treatment all patients underwent follow-up visits on a regular basis. These visits were scheduled every 3 months for the first 2 years, twice a year until the 5th year and yearly thereafter. For this study the follow-up information closeout date was December 2011 to guarantee a minimum follow-up of one year. A post-therapy baseline CT or MRI was performed eight to twelve weeks after the end of treatment. Time-to-event endpoints were calculated from end of IMRT until the date of event. Patients not experiencing an event were censored at the date of the last follow-up visit.

Toxicities were graded according to the National Cancer Institute (NCI) Common Terminology Criteria for adverse events (CTCAE) version 3.0. The symptoms of pain, dermatitis, mucositis, dysphagia, xerostomia and osteonecrosis were assessed. Acute and late toxicity were defined as post-treatment-related complications during and/or within 3 months after chemo-RT, and after 3 months, respectively. Baseline pre-treatment assessments using the same criteria were also performed.

### Analysis of locoregional failures

The earliest diagnostic imaging showing any LRF was matched to the planning CT and the treatment plan. All recurrent and/or persistent local or regional macroscopic tumor volumes (RTV) were then delineated on the planning CT and a quantitative comparison of the volume congruency was used to categorize the LRF as “in-volume”, if >95% of the RTV was included in the corresponding CTV54, CTV66 or CTV72; “marginal”, if 20–95%, and “out-volume”, if <20% of the RTV was included within the corresponding CTV, as previously described [16]. The relation of the RTVs to all CTVs was assessed sequentially. We decided to quantitatively compare RTVs to CTVs as opposed to treatment plan isodose lines [17] to better evaluate the accuracy of our target volume delineation and dose prescription.

### Statistical considerations

The primary endpoint was locoregional control (LRC). Secondary endpoints included local (LC) and regional (RC) controls, distant metastasis-free survival (DMFS), laryngectomy-free survival (LFS), overall survival (OS), acute and late toxicities. Late toxicity at last follow-up visit was also assessed to determine whether the late toxicity persisted or was transient. Time-to-event endpoints were estimated using the Kaplan-Meier method. Univariate and multivariate analyses were performed using Cox proportional hazards models. Known prognostic variables were analyzed in the multivariate analysis and a stepwise backward selection method (criterion for removal: p ≥0.05) was applied. Variables were summarized using absolute and relative frequencies. P-values were two-sided, not adjusted for multiple testing, and considered significant if < 0.05. The data were analyzed in SPSS (SPSS Inc., Chicago, IL, version 21.0).

## Results

### Patient and treatment characteristics

Of the 54 patients that met initial inclusion criteria and had started treatment, 3 did not finish treatment because of treatment-related complications (respiratory infection, aspiration, feeding tube insertion complication) and 1 had a total treatment time > 60 days, being excluded from final analysis. Finally, 50 patients were analyzed, 26 with hypopharyngeal cancers and 24 laryngeal cancers.

Pre-treatment diagnostic imaging of the head and neck was performed either by CT (n = 20), MRI (n = 21) or both (n = 9). Whole body staging consisted of a chest CT scan (n = 6), a PET-CT scan (n = 25) or both (n = 11). The remaining 8 patients had a chest radiograph.

Twenty-two patients (44%) underwent upfront ND, 13 of which were unilateral and 9 were bilateral. ECE was diagnosed histopathologically in 13 cases (10 ipsilateral only, 1 contralateral only, 2 bilateral). Two patients had a transoral debulking laser surgery to the primary tumor to improve breathing. Forty-three patients (86%) underwent either both neoadjuvant and concomitant (n = 7) or concomitant only (n = 36) systemic therapy. Concomitant therapy consisted of cisplatin (100mg/m2 three-weekly interval) in 25 cases (mean number of cycles 2.7; range 1-3), carboplatin (AUC5 three weekly interval or AUC2 weekly interval) in 7 cases or monoclonal antibody cetuximab in 9 cases (mean number of applications 6, range 1-8). Two patients started chemoradiation with cisplatin and continued with carboplatin because of toxicity. Seven patients were not deemed fit for any systemic therapy. Median RT total dose was 72 Gy (range, 66 - 74). One patient received 74 Gy to compensate for treatment interruption. Median treatment time was 51.7 days (range, 47 - 61), median follow-up for the surviving patients was 4.2 years (range, 1.0 – 6.6) and median follow-up for all patients was 3.2 years (range, 0.1 – 6.6). Further patient and treatment characteristics are summarized in Table 1.

**Table 1:** **Patient and treatment characteristics (n = 50)**

**Characteristics**	**n (%)**
Age (years)^β^	
≤60	19 (38))
≥60 to ≤ 70	20 (40)
>70 to ≤ 85	11 (22)
Gender	
Female	5 (10)
Male	45 (90)
Karnofsky PS	
>70	45 (90)
≤70	5 (10)
Site	
Hypopharynx: Post cricoid area	10 (20)
Hypopharynx: Piriform sinus	14 (28)
Hypopharynx: Posterior pharyngeal wall	2 (4)
Larynx: Supraglottis	14 (28)
Larynx: Glottis	10 (20)
Tumor classification	
cT1	2 (4)
cT2	5 (10)
cT3	27 (54)
cT4	16 (32)
Nodal classification	
cN0	11 (22)
cN1	8 (16)
cN2	11 (38)
cN3	2 (4)
pN1	2 (4)
pN2	15 (30)
pN3	1 (2)
Grading	
Moderate (G2)	32 (64)
Poor (G3)	18 (36)
Neck dissection^π^*	
None	28 (56)
Ipsilateral	13 (26)
Bilateral	9 (18)
Tracheotomy	
Before radiotherapy	7 (14)
During radiotherapy	9 (18)
After radiotherapy	3
Gastrostomy tube	
None	17 (34)
Used	33 (66)

*Abbreviations:* *PS* performance status, ^β^median 63 years (range, 45-84 years); ^π^Median of 56 nodes removed (range, 18 - 106 nodes); *Median time from neck dissection to radiotherapy was 42 days (range, 23 – 59 days); RT = Radiotherapy.

### Disease control, salvage surgery and patterns of failure

**Figure 1:**
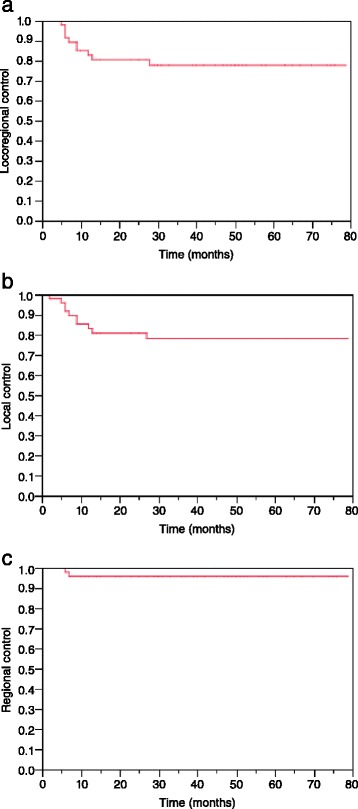
**Kaplan–Meier curves for locoregional control (a), local control (b) and regional control (c) for patients with locally advanced hypopharyngeal and laryngeal cancers treated with definitive IMRT.**

At the time of analysis 10 patients had developed LRF with the following characteristics: 8 unifocal local recurrences, 1 unifocal regional recurrence, 1 patient with combined local, multifocal regional and distant recurrence. One patient presumably died of tumor progression before restaging therefore this patient was not included in the detailed analysis of patterns of failure. The isolated regional recurrence occurred ispilaterally in a patient with ND prior to IMRT, and there was no contralateral regional failure. Median time to LRF was 0.8 years (range, 0.4 – 2.3). Kaplan-Meier estimates at 2 and 3 years for LRC were 80% and 77%, respectively (Figure 1a). Kaplan-Meier estimates at 2 and 3 years for LC and RC were 80% and 78% and 96% and 96%, respectively (Figure 1b-c). Neither in univariate nor in multivariate testing could a significant prognostic factor for LRC be identified (Table 2).

**Table 2:** **Treatment outcome analysis**

**Factor**	**Associated level**	**Cox regression analysis: hazard ratio (95% CI) (p-value)**	
		**LRC**	**OS**
*Univariate analysis*			
Age (years)	>60	0.86 (0.26, 2.817) (0.80)	1.96 (0.76, 5.07) (0.17)
Sex	Male	1.14 (0.15, 8.91) (0.90)	0.97 (0.23, 4.16) (0.96)
Localisation	Larynx	1.29 (0.39, 4.24) (0.67)	1.04 (0.44, 2.45) (0.94)
T-classification	cT_3-4_	1.29 (0.16, 10.06) (0.81)	0.53 (0.18, 1.58) (0.26)
N-classification	c/pN_2b-3_	1.13 (0.33, 3.87) (0.84)	0.78 (0.33, 1.83) (0.57)
AJCC stage	IV	0.90 (0.24, 3.40) (0.88)	0.91 (0.35, 2.35) (0.91)
Grading	G3	0.88 (0.26, 3.01) (0.84)	0.75 (0.30, 1.85) (0.53)
Neck Dissection	yes	1.48 (0.45, 4.86) (0.52)	0.19 (0.51, 2.81) (0.69)
Duration RT	>55 days	1.08 (0.23, 5.01) (0.92)	0.70 (0.21, 2.40) (0.57)
*Multivariate analysis*			
Age (years)	>60	0.93 (0.27, 3.39) (0.93)	2.37 (0.83, 6.73) (0.11)
Sex	Male	1.28 (0.13, 12.43) (0.83)	0.79 (0.14, 4.42) (0.79)
Localisation	Larynx	1.31 (0.33, 5.12) (0.70)	1.32 (0.46, 3.75) (0.60)
T-classification	cT_3-4_	1.51 (0.14, 10.79) (0.73)	0.50 (0.12, 2.05) (0.26)
N-classification	c/pN_2b-3_	1.26 (0.13, 12.32) (0.85)	0.55 (0.15, 2.02) (0.57)
AJCC stage	IV	0.51 (0.05, 5.38) (0.57)	0.92 (0.45, 8.15) (0.38)
Grading	G3	1.13 (0.28, 4.57) (0.87)	0.99 (0.36, 2.74) (0.98)
Neck Dissection	yes	1.83 (0.34, 9.89) (0.49)	1.54 (0.52, 4.55) (0.43)
Duration RT	>55 days	1.11 (0.22, 5.50) (0.90)	0.65 (0.17, 2.43) (0.52)

*Abbreviations:* *LRC* Locoregional control, *OS* Overall survival, *CI* confidence interval.

Diagnosis of LRF could be established within the first (n = 7), second (n = 2) or third year (n = 1) after completion of treatment. Salvage surgery was offered to 7 patients but performed in 6 patients (1 patient refused and underwent palliative chemotherapy). Only 1 patient profited at long-term being alive without evidence of disease (follow up time of 48 months post-surgery), and the remaining 5 patients suffered from incomplete resection, repeated recurrences and protracted postsurgical complications and finally died [median survival post salvage surgery of 7 months (range 3-24 months)]. Two patients underwent a second course of RT following salvage surgery.

LRF pattern analysis could be performed in 10 of the 11 patients. The RTVs were completely covered by the standard risk CTV54 in 9 of the 10 patients (90%) and 1 RTV was marginal to CTV54. RTVs were completely covered by the high-risk CTV66 and CTV72 in 6 out of 10 patients (60%) but 4 RTVs (40%) were marginal to the respective high-risk CTV. There was no out-volume failure (Table 3).

**Table 3:** **Patterns of recurrence according to CTV coverage**

**Patient**	**Primary tumor**	**Local vs. regional failure**	**Full coverage by CTV54**	**Marginal coverage by CTV54**	**Full coverage by CTV66**	**Marginal coverage by CTV66**	**Full coverage by CTV72**	**Marginal coverage by CTV72**	**Most probable explanation for relapse**
**1**	larynx	local	Yes	No	Yes	No	Yes	No	radioresistance
**2**	hypopharynx	local	Yes	No	NA	NA	No	Yes	underdosage
**3**	hypopharynx	local	Yes	No	Yes	No	Yes	No	radioresistance
**4**	larynx	local	Yes	No	Yes	No	No	Yes	underdosage
**5**	larynx	local	Yes	No	NA	NA	Yes	No	radioresistance
**6**	hypopharynx	regional	Yes	No	No	Yes	No	Yes	underdosage
**7**	larynx	local	Yes	No	NA	NA	Yes	No	radioresistance
**8**	larynx	local and regional	Yes	No	NA	NA	Yes	No	radioresistance
**9**	hypopharynx	local	Yes	No	No	Yes	No	Yes	underdosage
**10**	hypopharynx	local	Yes	No	Yes	No	Yes	No	radioresistance

*Abbreviations:* *CTV* clinical target volume, *CTV54* clinical target volume covered by 54Gy, *CTV66* clinical target volume covered by 66Gy, *CTV72* clinical target volume covered by 72Gy, *NA* non-applicable.

### Distant metastasis and overall survival

**Figure 2:**
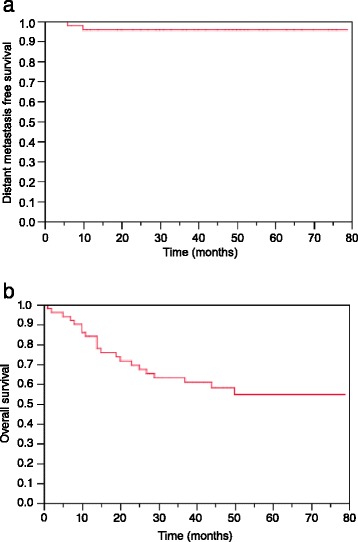
**Kaplan–Meier curves for distant metastasis free survival (a) and overall survival (b) for patients with locally advanced hypopharyngeal and laryngeal cancers treated with definitive IMRT.**

At the time of analysis, 2 patients developed distant metastasis, located in the lung (n = 1) and soft tissue and lung (n = 1). One patient with metastasis was simultaneously diagnosed with LRF. Median time to distant metastasis was 0.6 years (range, 0.5 – 0.8). Kaplan-Meier estimates at 2 and 3 years for DMFS were 96% and 96%, respectively (Figure 2a).

Twenty-one patients (42%) died during follow-up: 11 (22%) due to cancer progression including 10 of 11 patients with LRF and 1 with distant metastasis, 3 (6%) due to second malignancies, 5 (10%) due to preexisting medical co-morbidities (GI-bleeding, myocardial infarction, stroke, chronic obstructive airway disease, pulmonary embolism) and 2 (4%) due to unknown reasons. Kaplan-Meier estimates at 2 and 3 years for OS were 69% and 63%, respectively (Figure 2b). Neither in univariate nor in multivariate testing could a significant prognostic factor for OS be identified (Table 2).

### Pre-treatment morbidity, acute and late toxicity

Toxicity data including pre-treatment morbidity is presented in Table 4. Prior to RT start, 10 patients (20%) had grade 2 pain, 4 (8%) had grade 2 dysphagia and 3 (6%) had grade 3 dysphagia.

**Table 4:** **Pre-treatment morbidity and acute and late toxicity**

		**Pre-Tx**	**Acute** ^**†**^	**Late** ^**‡**^	**Last late** ^**§**^
**Toxicity**	**Grade**	**n (%)**	**n (%)**	n (%)**	n (%)**
Pain	0	24 (48)	-	36 (77)	42 (90)
	1	16 (32)	3 (6)	5 (11)	2 (4)
	2	10 (20)	34 (68)	4 (8)	1 (2)
	3	-	13 (26)	2 (4)	2 (4)
Dermatitis	0	50 (100)	1 (2)	46 (98)	47 (100)
	1	-	5 (10)	1 (2)	-
	2	-	31 (62)	-	-
	3	-	13 (26)	-	-
Mucositis	0	50 (100)	3 (6)	46 (98)	47 (100)
	1	-	8 (16)	1 (2)	-
	2	-	31 (74)	-	-
	3	-	8 (16)	-	-
Dysphagia	0	20 (40)	1 (2)	28 (60)	37 (78)
	1	23 (46)	5 (10)	7 (15)	5 (11)
	2	4 (8)	27 (54)	8 (17)	5 (11)
	3	3 (6)	17 (34)	4 (8)	-
Xerostomia	0	50 (100)	37 (74)	23 (49)	40 (85)
	1	-	9 (18)	21 (45)	6 (13)
	2	-	4 (8)	3 (6)	1 (2)
	3	-	-	-	-
Osteonecrosis	0	50 (100)	49 (98)	45 (96)	46 (98)
	1	-	-	-	-
	2	-	-	-	-
	3	-	1 (2)	2 (4)	1 (2)
Highest*	0	13 (26)	-	15 (32)	30 (64)
	1	20 (40)	-	14 (30)	9 (19)
	2	14 (28)	19 (38)	11 (23)	6 (13)
	3	3 (6)	31 (62)	7 (15)	2 (4)

*Abbreviations:* *Pre-Tx* pre-treatment morbidity, *The highest morbidity/toxicity in a patient was counted as a single event; ^†^During therapy and until 3 months after completion; ^‡^Maximal late toxicity > 3 months after completion of therapy; ^§^Incidence of late toxicity at last follow-up visit. **3 patients died within the acute toxicity period.

The highest-grade acute toxicities were grade 2 in 19 patients (38%) and grade 3 in 31 patients (62%). There were no grade 4 or 5 toxicities, and no treatment was interrupted due to toxicity. The highest-grade late toxicities were grade 2 in 11 patients (23%) and grade 3 in 7 patients (15%). At the last follow-up 6 patients (13%) still presented grade 2; and 2 patients (4%) presented grade 3 toxicity. Of note, highest-grade toxicity rates at the last follow up were inferior to the pre-treatment morbidity rates (Table 4).

### Feeding tube insertion and removal

Feeding tube insertion via percutaneous endoscopic gastrostomy (PEG) was generally recommended, though 24 (48%) patients refused it. PEG was finally performed prior to start of IMRT in 26 (52%) patients, during IMRT in 5 (10%) patients and in 2 (4%) patients after LRF (9 and 10 months after completion of treatment, respectively). A total of 19 (38%) patients were irradiated without feeding tube support. Median time to feeding tube removal was 8 months (range, 1-25). All 29 living patients were without feeding tube at last follow-up visit and had regular oral nutritional intake.

### Tracheostomy tube insertion and salvage laryngectomy

**Figure 3:**
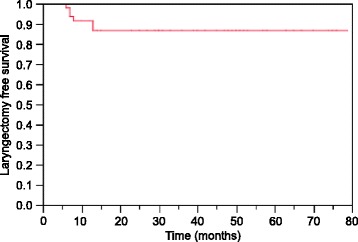
**Kaplan–Meier curves for laryngectomy free survival of patients with locally advanced hypopharyngeal and laryngeal squamous cell carcinoma treated with definitive IMRT.**

A total of 9 patients needed a tracheostoma to be able to receive treatment; tracheostomy was performed prior to IMRT start in 8 patients and during IMRT in 1 patient. Another 3 patients had a tracheostomy because of LRF after treatment completion. A total of 6 laryngectomies were performed, all in the setting of surgical salvage after LRF. No laryngectomy was performed because of chondronecrosis or dysfunction. At the last follow-up, 28 of 29 living patients were free of tracheostomy tubes with a functional larynx. The 2 and 3 year LFS estimates were 86% and 86%, respectively (Figure 3).

## Discussion

The majority of publications reporting IMRT outcomes in head and neck cancers include an inhomogenous patient selection concerning primary site, stage and definitive or postoperative use of IMRT [11,17-22]. In the past years a growing number of studies have reported definitive IMRT outcomes for HL-SCC as summarized in Table 5.

**Table 5:** **Selected series of hypopharygeal and laryngeal cancer patients treated with definitive IMRT**

**Study/Year**	**N**	**Site of primary**	**AJCC Stage**	**Median FU in months (range)**	**PTV-HD dose prescription**	**Margin GTV to PTV-HD**	**LRC**	**OS**
**Miah 2012** [23]		HC + LC	II: 2			23 mm or entire organ + 3 mm		
	Grp1: 29		III: 28	51 (12-77)	Grp1: 63Gy/28f		Grp1: 67%, 2y	72%, 2y
	Grp2: 31		IV: 30	36 (04-63)	Grp2: 67Gy/28f		Grp2: 82%, 2y	74%, 2y
**Nguyen 2012** [24]	27	LC	III: 17	20 (6-57)	70Gy/35f	5-10 mm	n.r.	80%, 2y
			IV: 10					62%, 3y
**Liu 2010** [22]	27	HC	II: 5	36 (2-82)	72.6Gy/35f	5 mm	63%, 5y	35%, 5y
			III: 4		76.8Gy/37f			
			IV: 18					
**Huang 2010** [25]	33	HC	II: 2	25.8 (14.2-72.3)	70Gy/35f	4 mm	n.r.	44%, 5y
			III: 5					
			IV: 26					
**Studer 2010** [26]	123	HC+LC	II: 16	26 (3-83)	66Gy/30f	10-15 mm	77%, 2y	83%, 2y
			III: 23		69.6/33f			
			IV: 77		70Gy/35f			
**Daly 2011** [27]	31	HC+LC	II: n.r.	30 (13-98)	66Gy/30f	8-13 mm	80%, 3y	46%, 3y
			III: n.r.					
			IV: n.r.					
**Mok 2014** [28]	181	HC	n.r.	IMRT: 50.4	60Gy/25f	5-10 mm	IMRT: 75%, 3y	IMRT: 50%, 3y
				3D-RT: 106.8	62Gy/40f			
					64Gy/40f		3D-RT: 58%, 3y	3D-RT: 52%, 3y
					70Gy/35f			
**Lee 2007** [29]	31	HC+LC	III: 9	24 (17-58)	70Gy/33f	5-10 mm	LRPFS	63%, 2y
			IV: 21				84%, 2y	
**Current study**	50	HC+LC	III: 14	39 (1-79)	70Gy/35f	8-15 mm	80%, 2y	69%, 2y
			IV: 36				77%, 3y	63%, 3y

*Abbreviations:* *N* number of patients, *AJCC* American Joint Commission on Cancer, *FU* Follow-Up, *PTV-HD* Planning Target Volume High-Dose, *GTV* Gross Tumor Volume, *LRC* loco-regional control, *OS* overall survival, *Grp1* Group treated with moderate acceleration, *Grp2* Group treated with dose escalation, *HC* Hypopharyngeal cancer, *LC* Laryngeal Cancer, *n.r.* not reported.

This work reports our single institution experience that includes treatment results of a well-defined cohort of 50 locally advanced HL-SCC patients treated with IMRT with an analysis of RTVs in relation to corresponding CTVs. Our RT fractionation (single and total doses), PTV dose prescription and concurrent chemotherapy regimen are well established in literature [3]. Although two patients underwent debulking procedures of the primary lesion we would still call it “definitive” or primary IMRT, as these two surgeries were not radical resulting in gross residual disease, thus the same principles for CTV definition to the primary were applied. On the other hand, anatomical changes due to upfront ND performed in 23 patients required adaptations in the CTV delineation of the neck in accordance to the specific clinical settings. Although this could have been a source of heterogeneous outcomes, CTV contouring followed standardized guidelines and important deviations were not seen.

Overall, our long-term LRC and OS rates are comparable with previously published outcome data following definitive IMRT treatment for locally advanced HL-SCC (Table 5). Of note, our experience confirms the importance of the primary treatment for effective LRC due to the very limited results achieved with salvage surgery. Although there are few data on LFS published, our LFS estimates were in the range of recent publications [28]. Detailed analysis of LRF revealed 4 marginal failures (3 local and 1 regional), where the RTVs were not fully covered by the high-dose CTV66 or CTV72 (Table 3). The isolated regional failure occurred in a patient with bilateral ND, tracheostomy and bilateral ECE and was located at the medial caudal edge of the right neck with infiltration of the thyroid gland. Compared to the treatment plan, about half of the RTV was outside the identifiable surgically manipulated area of the ND and thus not covered by the CTV66. Intraoperative seeding of tumor cells might explain the unusual location; however we did not see how this could have been accounted for at the time of target volume definition. The remaining three marginal failures were isolated local failures; all three had advanced T3/T4 primaries. Perhaps an underestimation of the GTV extent might have contributed to the marginal local failures as two patients were initially staged with CT and PET-CT, but did not have MRI (one patient had claustrophobia). The GTV to PTV margin was at the lower end of our range with the intention to spare dose to pharyngeal constrictor muscles. Based on the described experience, we have meanwhile introduced additional locoregional MRI imaging for all locally advanced cases and discussed expansion of the minimum GTV to PTV high-risk margin in selected cases especially when the primary tumor is ill-defined with difficult GTV delineation. Our target volume definition for the standard risk CTV54 seemed to be adequate with only one of 10 RTV marginal to CTV54 and the rest completely in-volume. Overall, neck control was high with only 2 regional failures and a 2-year regional control of 96%. A higher number of regional failures outside high dose volume could be explained by limitations in clinical lymph node staging. Our low rates of regional failures could be partially the result of the therapeutic effect of upfront ND providing improved pathologic staging in selected cases prior to definitive IMRT. Upfront ND offers encouraging LRC and survival rates in retrospective cohorts [30] however its definite role still warrants validation in randomized trials.

There are different methods for assessing and classifying RTVs in relation to the initial treatment plan, being challenging to thoroughly compare patterns of failure between publications. Dawson et al. [17] compared the RTV to the respective 95% prescription isodose line of the treatment plan, and Chao et al. [16] compared the RTV to the respective CTV. Both defined marginal recurrence if more than 5% of the RTV is outside of the respective 95% isodose line or CTV, respectively. In contrast, other authors [21] defined marginal recurrence if more than 50% of the RTV was outside the respective 95% isodose line. If we had used this definition, we would have reported only one RTV marginal to the high-risk CTV with 9 of 10 (90%) RTV fully covered. When classifying RTV as “in-volume”, “marginal” or “out-volume”, additional important data should be provided as which CTV (high- or standard-risk) or which dose prescription was used as reference. In some publications regional recurrences fully included in the elective nodal target volume and having been irradiated with the elective dose are reported as “in-volume” recurrence only. We think that these recurrences are indeed “in-volume” to the standard risk CTV but “out-field” to the high-risk CTV thus should be classified as such, indicating that the dose prescription to the RTV region was not sufficient.

Our cohort showed that although there were marginal recurrences potentially explained by an underdosage of the tumor, radioresistant clones continue to be a major barrier in the treatment of head and neck tumors. Altered RT fractionation (e.g. accelerated regimens) can shorten the overall treatment duration, thereby attempting to minimize tumor repopulation as a cause of treatment failure. Nevertheless the available data suggest that accelerated RT regimens do not improve OS when given with concurrent chemotherapy and/or cetuximab [31,32]. Numerous molecularly targeted agents directing specific cell signaling pathways involved with radioresistance (e.g. VEGF, DNA repair, apoptosis, hypoxia, and proliferation) have been tested in preclinical and clinical studies [33]. In addition, novel strategies to enhance patients’ stratification using molecular and genetic signatures along with HPV status as biomarkers of treatment response need to be further explored and timely incorporated into the decision-making process.

## Conclusion

Our study confirms that definitive, conventionally fractionated IMRT in locally advanced HL-SCC results in good LRC rates with acceptable toxicity profile. Our pattern of failure analyses shows predominantly local failures with high rates of regional and distant control. Although the recurrences marginal to the high-risk CTV imply that optimization in RT volumes could improve outcomes, in-volume relapses are the major type of recurrence, which underlines the need for better strategies to overcome tumor radioresistance.
